# Use of speckle-tracking strain in preload-dependent patients, need for cautious interpretation!

**DOI:** 10.1186/s13613-018-0376-8

**Published:** 2018-02-21

**Authors:** C. Nafati, M. Gardette, M. Leone, L. Reydellet, V. Blasco, A. Lannelongue, F. Sayagh, S. Wiramus, F. Antonini, J. Albanèse, L. Zieleskiewicz

**Affiliations:** 1 Department of Anesthesia and Intensive Care Medicine, University Hospital of Marseille, la Timone Hospital, Marseille, France; 2 Department of Anaesthesia and Intensive Care Medicine, University Hospital of Marseille, North Hospital, Marseille, France; 3 Centre d’Investigation Clinique, Aix-Marseille University, AP-HM, 14901 Marseille, France; 4 Service d’anesthésie et de réanimation, CHU de la Timone, 264 rue Saint Pierre, 13005 Marseille, France

**Keywords:** Preload dependence, Fluid responsiveness, Passive leg raising, 2D-strain echocardiography, Speckle tracking

## Abstract

**Background:**

In critical patients, left ventricular ejection fraction and fractional shortening are used to reflect left ventricular systolic function. An emerging technique, two-dimensional-strain echocardiography, allows assessment of the left ventricle systolic longitudinal deformation (global longitudinal strain) and the speed at which this deformation occurs (systolic strain rate). This technique is of increasing use in critical patients in intensive care units and in the peri-operative period where preload constantly varies. Our objective, in this prospective single-center observational study, was to evaluate the effect of fluid resuscitation on two-dimensional-strain echocardiography measurements in preload-dependent critically ill patients. We included 49 patients with preload dependence attested by an increase of at least 10% in the left ventricular outflow track velocity–time integral measured by echocardiography during a passive leg raising maneuver. Echocardiography was performed before fluid resuscitation (echocardiography 1) and after preload independency achievement (echocardiography 2).

**Results:**

Two-dimensional-strain echocardiography was feasible in 40 (82%) among the 49 patients. With preload dependence correction, the absolute value of global longitudinal strain and systolic strain rate was significantly increased from, respectively, − 13.3 ± 3.5 to − 18.4% ± 4.5 (*p* < 0.01) and − 1.11 s^−1^ ± 0.29 to − 1.55 s^−1^ ± 0.55 (*p* < 0.001). The fluid resuscitation affects GLS and SSR in preload-dependent patients, with a shift, for GLS, from pathological to normal values.

**Conclusion:**

In critically ill patients, the assessment of the systolic function by two-dimensional-strain echocardiography needs prior evaluation of preload dependency, in order to adequately interpret this variable. Future studies should assess the ability of global longitudinal strain to guide fluid management in the critically ill patients.

## Background

Systolic function assessment is crucial in the management of the critically ill patient. Using conventional echocardiography, left ventricular ejection fraction (LV EF) and fractional shortening are routinely used [1]. However, these variables depend on preload and afterload conditions [2]. They can be difficult to interpret in unstable patients such as those in septic shock [3].

Strain echocardiography (2D-strain) is a noninvasive ultrasound imaging technique that allows for an objective and quantitative evaluation of myocardial function. It measures the percentage of deformation of the left ventricle (LV) during systole (systolic strain) and the speed at which this deformation occurs (strain rate) [4, 5]. This technique has been validated after comparison with reference techniques: magnetic resonance imaging and sonomicrometry [5, 6]. The American Society of Echocardiography and the European Association of Cardiovascular Imaging recommended measuring the Global Longitudinal Strain Systolic in apical 2-, 3- and 4-chamber views (GLS) using 2D-strain to evaluate the LV systolic function [7]. Normal values are below − 18% in healthy subjects [8, 9].

In critically ill patients, 2D-strain allows early diagnosis of cardiac injuries that are non-detectable with conventional examinations [10, 11]. Previous studies identified GLS as an independent factor of mortality [12, 13]. In the peri-operative setting, speckle tracking is an emerging tool for the early detection of right and left ventricular dysfunction [14]. Kovács et al. [15] found that 2D-strain reflects inotropism and correlates with elastance (*E*_max_) and intraventricular pressure–volume curves in a rat model. In cardiology patients undergoing routine coronary angiography, fluid resuscitation did not affect GLS and systolic strain rate (SSR) [16]. Other studies [17, 18] in normal volunteers found diverging results. Conversely, animal studies suggest that fluid loading or acute unloading affects GLS and SSR [19, 20]. One clinical study in postcardiac surgery patients finds the same results, showing that the GLS and SSR are dependent on preload condition [21]. If for GLS, preload dependence is accepted, for the SSR this remains controversial. We hypothesized that this discrepancy was due to the position of the patient on the Frank and Starling curve. In the critically ill patient, preload and afterload constantly vary and preload dependency is an important parameter concerning up to 50% of these patients [22, 23]. The goal of our study was to evaluate for the first time in the ICU, the effect of fluid resuscitation on GLS and SSR in preload-dependent critically ill patients.

## Methods

Our study was a prospective observational study carried out in a single 12-bed polyvalent ICU at the University Hospital La Timone in Marseille, from August to December 2016. The protocol was approved by the Ethics Committee of the French Society of Anesthesiology and Intensive Care (IRB 00010254-2016-078) on August 18, 2016. Data collection was authorized by the CNIL (French Data Protection Authority, Receipt No. 1995927v0). Exclusion criteria were: patient under 18 years of age, absence of normal sinus rhythm, patient and/or person of trust’s refusal to participate in the study.

**Fig. 1:**
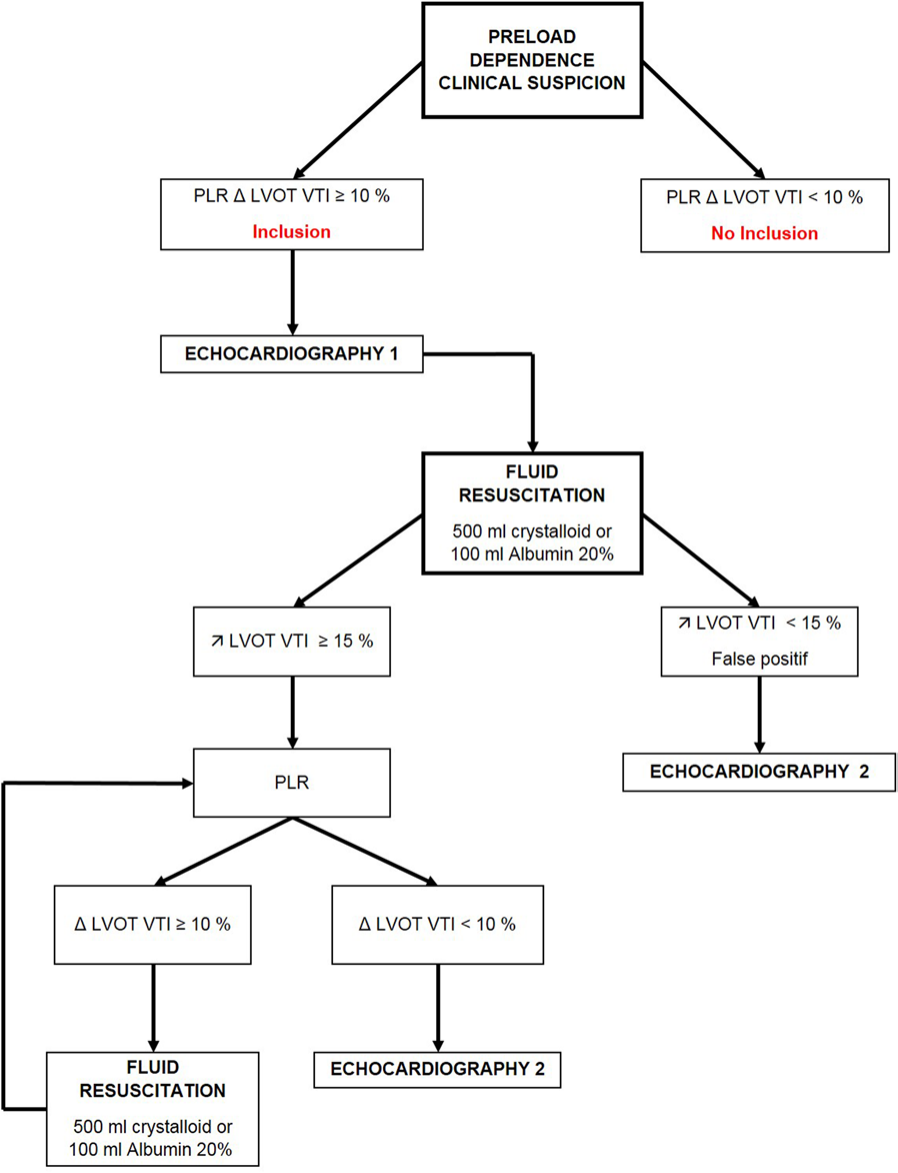
Flow chart protocol. PLR, passive leg raising; PLR Δ LVOT VTI, passive leg raising variation of left ventricular outflow track velocity–time integral; ↗ LVOT VTI, increase of left ventricular outflow track velocity–time integral after fluid resuscitation

We included all patients with preload dependence. Patients in acute circulatory failure were also included, if they were preload dependent. First, preload dependency was suspected according to at least one of the following criteria: hypotension or/and oligo-anuria or/and difficulty in weaning of catecholamine. Second, preload dependence was confirmed by an increase of at least 10% of the left ventricular outflow track velocity–time integral (Δ LVOT VTI ≥ 10%) measured by echocardiography during a passive leg raising (PLR) [24]. If preload dependence was confirmed, the first echocardiogram was performed (echocardiography 1) with conventional and 2D-strain measurements. Then fluid resuscitation was started as follows: 500 mL of crystalloids administered over 30 min or 100 mL of 20% albumin administered over 60 min. The patient was considered as responsive if this fluid challenge was followed by a 15% increase in the LVOT VTI, and the patient was otherwise considered non-responsive [25]. The patient was considered as non-preload-dependent if the change in the (Δ) LVOT VTI during the PLR was below 10%. The fluid resuscitation could be repeated if needed, after checking the persistence of preload dependence criteria. When the patient was no longer preload dependent (Δ LVOT VTI PLR < 10%), a second echocardiogram was performed (echocardiography 2) with conventional and 2D-strain measurements. The choice of the resuscitation fluid (crystalloid or albumin 20%) was determined by the attending physician (Fig. 1).

Acute circulatory failure was defined as mean arterial pressure < 65 mmHg, urine output < 0.5 ml/kg/h, mottled skin or arterial blood lactate > 2 mmol/L [26].

The features (age, sex, weight, height, reason for admission), the cardiovascular comorbidities, SAPS II, SOFA and the arterial lactate levels were recorded for each patient included in the study. The arterial pressures (systolic, diastolic and mean) and heart rate were recorded before and after each fluid resuscitation. Mortality at 28 days was recorded by consulting the electronic patient files.

### Echocardiography protocol

Once the preload dependence was confirmed, two echocardiography studies were performed, the first one before fluid resuscitation (echocardiography 1) and the second one after, when the patient was no longer preload dependent (echocardiography 2). Both were performed by an expert certified physician (CN) and using a Philips CX 50 Compact Xtreme Ultrasound System (Philips Medical System. Andover. MA) and a 3.5-Hz probe. Conventional and 2D-strain echocardiography was used in each study.

The conventional evaluation was performed following the American Society of Echocardiography Recommendations [7]. The bi-dimensional measures were taken using a parasternal long-axis view with a motion (*M*)-mode to determine the right ventricle size, interventricular septal wall thickness, left ventricle size, ratio of right ventricle/left ventricle and the diameter of the LV outflow tract. The tricuspid annular plane systolic excursion (TAPSE) was measured from an apical 4-chamber view with M-mode. We visually verified using an apical 4-chamber view that the RV/LV ratio was < 1. The LVOT VTI was measured using pulse-wave Doppler from an apical 5-chamber view. Mitral flow was assessed using an apical 4-chamber view with pulse-wave Doppler allowing measurement of the *E-* and *A*-wave velocities, the *E/A* ratio and the *E*-wave deceleration time.

Using tissue Doppler imaging from an apical 4-chamber view, the velocity of the septal and lateral mitral annulus *E*′ waves was measured (*E*′_lateral_ and *E*′_medial_), and *E*′_median_ (average between *E*′_lateral_ and *E*′_medial_), *E*/*E*′_lateral_, *E*/*E*′_medial_ and *E*/*E*′_median_ ratios were calculated. The ejection fraction was measured using the Simpson’s biplane method in a 4- and 2-chamber view. All recorded values were averaged using three measurements.

With regard to the 2D-strain, a 2-s loop was recorded in DICOM format for each view with a frame rate above 50/s. The analysis and measures of strain and strain rate were performed off-line using the QLAB Philips software (Philips Medical System Andover, MA, USA) by a level 3 [27] operator (C.N.) fully trained in 2D-strain echocardiography. Particular attention was given to obtain an adequate grayscale image, allowing reliable delineation of myocardial tissue. The left ventricular myocardial contour was traced using the semiautomatized method of speckle tracking after identification of baso-septal, baso-lateral and apical points [28]. Adequate tracking can be verified in real time from the dynamic loop and manually adjusted if needed. The GLS was calculated as the average speckle-tracking systolic peak of strain from each of 18 LV segments from the apical 4-chamber view, 2-chamber view and 3-chamber view as recommended [7]. The SSR was the most negative value of the strain rate curve occurring after the opening of the aortic valve. The measure of GLS was considered successful if at least the 4-chamber systolic strain (S4C) and one or both strain among the 2 (S2C)- and 3-chamber view (S3C) were obtained [8]; otherwise, the LV was considered to have insufficient image quality. Segmental data were not analyzed. Strain and strain rate are negative values; the more negative the value is, the greater the deformation and LV function are. GLS and SSR were considered decreased when GLS > − 15% and SSR > − 1 s^−1^ [29].

### Statistical analysis

All analyses were performed using R-Project for Statistical Computing 2.14 (The R Foundation, Vienna, Austria). Categorical variables were expressed as numbers and percentages (%). Continuous variables were expressed as mean ± SD or median and interquartile range (25th–75th) depending on their distribution. The Kruskal–Wallis test, the Fisher exact test and the ANOVA test *r*^2^ were used to compare the distribution of variables. In univariate analysis, factors were considered significant when *p* < 0.05. We calculated that at least 38 patients would need to be enrolled to detect a 4% increase in GLS after fluid expansion with a 90% statistical power and a two-sided alpha value of 0.05. In order to exclude the patients in whom cardiac ultrasound was not technically feasible, we decided to include 49 patients. The correlation between the global longitudinal strain and the 4-chamber strain was performed using Spearman’s method. From all patients, intraoperator variability of GLS was determined by the intraclass correlation coefficient.

## Results

Forty-nine patients were included over the period of August–December 2016.

### Clinical characteristics of study patients

The patients’ clinical characteristics are presented in Table 1. Preload dependency was suspected because of hypotension in 29 (59%) patients, anuria in 8 (16%) patients and due to difficult catecholamine weaning in 12 (25%) patients.

**Table 1:** Clinical characteristics of study patients

Total number of patient (*n*)	49
Sex ratio (m/w)	29/20
Age (years)	64 ± 15
*BMI* (kg/cm^2^)	25 ± 5
Mechanical ventilation [*n* (%)]	18 (36)
Vasopressor [*n* (%)]	22 (45)
Lactate	2.9 ± 3.9
SAPS II	51 ± 17
SOFA	7.2 ± 3.5
28 Days mortality rate [*n* (%)]	11 (22)
Cardiovascular comorbidities	
Arterial hypertension	18 (37)
Coronary disease	5 (10)
Arrhythmia	1 (2)
Valvular disease	1 (2)
Reason for admission in ICU [*n* (%)]	
Septic shock	16 (32)
Hemorrhagic shock	6 (12)
Cardiogenic shock	1 (2)
Surgery	15 (30)
Cardiac arrest	2 (4.10)
Acute pancreatitis	1 (2)
Myasthenia	1 (2)
Hepatitis	2 (4.1)
Suicide	2 (4.1)
Liver transplantation	3 (6.1)

Data are expressed as number (%) or mean ± SD

*BMI* body mass index, *SAPS* Simplified Acute Physiology Score, *SOFA* Sequential Organ Failure Assessment, *ICU* intensive care unit

### Ultrasound data

Data are shown in Table 2. For 9 (18%) patients, the echocardiography quality was insufficient for strain measurement resulting in analysis of data in 40 patients. The average frame rate was 60/s ± 1.6.

**Table 2:** Echocardiography and clinical data before and after fluid resuscitation

	Before	After	*p* value
Heart rate (p/m)	103 ± 20	97 ± 15	0.18
SAP (mmHg)	95 ± 17	119 ± 14	< 0.001
DAP (mmHg)	50 ± 12	57 ± 10	0.01
MAP (mmHg)	65 ± 13	77 ± 10	< 0.001
*E* (cm/s)	73 ± 23	91 ± 26	0.003
*E*/*A*	0.9 ± 0.3	1.0 ± 0.3	0.13
DTE	200 ± 16	200 ± 12	0.78
*E*′_SEPT_ (cm/s)	13.6 ± 3.7	12.9 ± 3.4	0.36
*E*′_LAT_ (cm/s)	14.2 ± 3.6	14.3 ± 3.5	0.79
*E*/*E*′_SEPT_	5.8 ± 2	7.3 ± 2.4	0.004
*E*/*E*′_lat_	5.4 ± 1.9	6.7 ± 2.4	0.001
*E*/*E*′_moy_	5.5 ± 1.9	6.9 ± 3.3	0.005
LVOT VTI (cm)	15.6 ± 3.7	21.1 ± 4.5	< 0.001
CO (L/min)	4.4 ± 1.8	5.7 ± 5.7	0.007
CI (L/min/m^2^)	2.4 ± 0.9	3.1 ± 1.2	0.007
LV EF (%)	61.3 ± 15.9	60.7 ± 15	0.78
LVOT diameter (cm)	1.8 ± 0.1	1.8 ± 0.1	0.89
LVTDV (mL)	104.5 ± 29	131 ± 34	< 0.001
RV/LV	0.6 ± 0.1	0.6 ± 0.1	0.62
TAPSE (mm)	22 ± 7	23 ± 5	0.87
SSR (s^−1)^	− 1.1 ± 0.29	− 1.55 ± 0.55	< 0.001
GLS	− 13.3 ± 3.5	− 18.4 ± − 4.5	< 0.001
S4C (%)	− 13.5 ± 4.1	− 18.7 ± 4.9	< 0.001
S2C (%)	− 13.4 ± 3	− 18.4 ± 4.8	< 0.001
S3C (%)	− 12.6 ± 3.7	− 18.4 ± 4.8	< 0.001

Data are expressed as mean ± SD

*SAP* systolic arterial pressure, *DAP* diastolic arterial pressure, *MAP* mean arterial pressure, *E* peak early diastolic transmittal flow velocity, *E/A* ratio of e to a, *TDE* E-wave deceleration time, *E*′_l_ peak early diastolic lateral mitral annulus velocity, *E*′_s_ peak early diastolic septal mitral annulus velocity, *E/E*′ ratio of *E* to *E*′, *LVTO VTI* left ventricular outflow tract velocity–time integral, *CO* cardiac output, *LV EF* left ventricle ejection fraction, *TAPSE* tricuspid annular systolic excursion, *LVDV* left ventricular tele-diastolic volume, *SSR* systolic strain rate, *GLS* global longitudinal strain, *S4C* 4-chamber systolic strain, *S2C* two-chamber systolic strain, *S3C* three-chamber systolic strain

### Effect of fluid resuscitation on standard clinical and echocardiographic values

Crystalloid, albumin or both were administered to 25 (51%) patients, 15 (30%) patients and 9 (19%) patients, respectively. The volume of crystalloid and albumin was 960 ± 310 and 256 ± 51 mL, respectively.

There was a significant increase in systolic arterial pressure (SAP), diastolic arterial pressure (DAP) and mean arterial pressure (MAP) after fluid resuscitation. There was also a nonsignificant decrease in heart rate (HR).

**Fig. 2:**
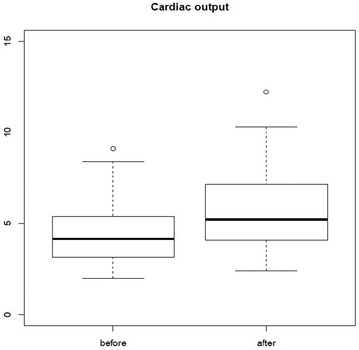
Significant increase of cardiac index before and after fluid challenge 2.4 ± 0.9–3.1 ± 1.2 L/min/m^2^ (*p* < 0.01)

Out of the 49 preload-dependent patients, 48 patients increased their cardiac output after fluid resuscitation by at least 15%. The LVOT VTI increased from 15.6 cm ± 3.7 to 21.1 cm ± 4.5 before and after fluid resuscitation (*p* < 0.01). After fluid resuscitation (*p* < 0.01), cardiac index increased from 2.4 ± 0.9 to 3.1 ± 1.2 L/min/m^2^ (*p* < 0.01) (Fig. 2). We observed a significant increase in left ventricular end-diastolic volume (LVEDV): 104.5 ± 29–131 ± 34 mL (*p* < 0.001). We did not find a significant change in the LV EF after fluid resuscitation. In our study, no patients had right heart failure.

After fluid resuscitation, *E*-wave significantly increased from 73 ± 23 to 91 ± 27 cm/s (*p* < 0.01). *E*/*E*′_lateral_, *E*/*E*_medial_ and *E*/*E*_median_ increased from 5.4 ± 1.9 to 6.7 ± 2.4 (*p* < 0 0.01), 5.8 ± 2 to 7.3 ± 2.4 (*p* < 0.01) and 5.5 ± 1.9 to 6.9 ± 2.3 (*p* < 0.01).

### Effect of fluid resuscitation on strain and strain rate values

**Fig. 3:**
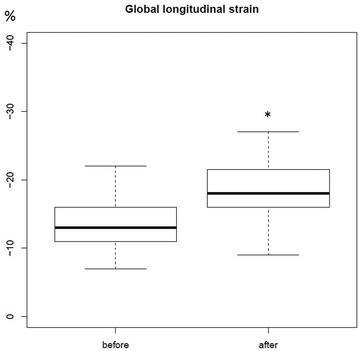
Significant increase of absolute value in GLS: − 13.3 ± 3.5 versus − 18.4 ± 4.5% (*p* < 0.01)

**Fig. 4:**
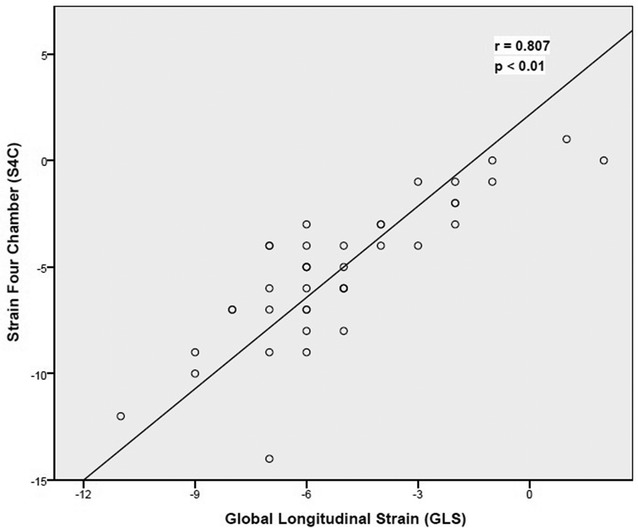
Correlation of global longitudinal strain and strain 4 chamber *r* = 0.81 (*p* < 0.01)

After fluid resuscitation, the absolute value of GLS significantly changed from − 13.3% ± 3.5 to − 18.4% ± 4.5 (*p* < 0.01) (Fig. 3). This variation was confirmed by the absolute strain values measured in the four-chamber (14% ± 4.1 vs. 19% ± 4.9), three-chamber (13% ± 3.5 vs. 18% ± 4.3) and two-chamber (13% ± 3.7 vs. 18% ± 4.8) views (*p* < 0.01). The GLS and the 4-chamber view strain values had good correlation (*r* = 0.81, *p* < 0.01) (Fig. 4). Regarding the SSR, the absolute value of SSR significantly changed from − 1.11 s^−1^ ± 0.29 to – 1.55 s^−1^ ± 0.55 (*p* < 0.001).

The intraclass correlation coefficient of the intraoperator variability of GLS was 0.92 (*p* < 0.001).

## Discussion

The main result of our study was to show that fluid resuscitation affects GLS and SSR in preload-dependent patients, with a shift, for GLS, from pathological to normal values.

Hence, in the critically ill patient, pathological values of GLS should be interpreted with prudence.

Recently, Boissier et al. [30] showed that GLS was inversely correlated with afterload. They did not find a correlation between preload and GLS values. However, based on the low collapsibility index of the superior vena cava reported in their patients, preload dependency was unlikely. Certain studies do not find modification of the SSR [17, 31] and/or of the GLS [18, 31] following preload variations. In these studies, this can be explained by the population consisting of healthy conscious volunteers (Abali et al. and Anderson et al.), the preload variation being smaller than that observed in the ICU patient and that the patients were not necessarily in a preload dependency state (Mendes et al.). Furthermore, these variations would activate baroreceptor-mediated counter-regulatory changes in the sympathetic outflow to the heart in such a population. This physiologic response was probably insufficient in our population of preload-dependent patients. Recently, Fredholm et al. [21] in a clinical study of postoperative cardiac surgery found similar results. They conclude that GLS and SSR were preload dependent. We explain the significant increase in the GLS and SSR, after preload correction, by the increased end-diastolic stretch of the left ventricle fibers (a significant increase in LVEDV 104.5 ± 29 mL to 131 ± 34, *p* < 0.001). The patients being in the preload-dependent zone are on the steep portion of the Franck–Starling curve. The preload increase then leads to an increase in the contraction force and velocity, as shown by Sonnenblick et al. in an experimental study in 1962 [32]. This does not occur in non-preload-dependent patients. In our false-negative patient, GLS or SSR did not change after fluid resuscitation.

More specifically for the SSR, in our study, there was a significant increase with preload correction; however, in contrast with the GLS, its value in hypovolemic patients is not pathological (− 1.11 ± 0.29 s^−1^). We explain this by the fact that not only is SSR influenced by preload, but it is also influenced by the heart rate. These results are aligned with those of Fredholm et al., who found in their study that the SSR is both preload and pace dependent. We believe that the SSR staying in a physiologic zone is due to the tachycardia provoked by the hypovolemia (HR: 103 ± 20 in our study). This is, in our opinion, a physiologically adaptive mechanism to maintain a certain intrinsic contractility in hypovolemic patients. This phenomenon is known as the force–frequency relationship [33].

We are not surprised that the LV EF is around 60% in our preload-dependent cohort. Indeed, in these hypovolemic patients, the preload is lowered and the cardiac chambers are reduced in size. The LVESV is decreased and tends toward zero (kissing heart) which leads to an LV EF increase tending toward 100% (LV EF = LVEDV − LVESV/LVEDV × 100). Thus, in our hypovolemic patients, the LV EF is mathematically elevated. Furthermore, we had a high proportion of septic shock patients in our population (32%), and because of the vasoplegia intrinsic to this pathology, there were numerous hyperkinetic patients with a LV EF > 60%. Boissier et al. found, in their cohort of septic shock patients, 103 patients with normal or hyperkinetic LV EF out of 130 [30].

We did not find a significant increase in LV EF after preload correction, whereas we did find a significant increase in LVEDV and GLS. We explain this because the LV EF and GLS are not comparable. The GLS formula *L* *−* *L*_*o*_/*L*_*o*_ (*L*: the length of contracted myocardial fibers, *L*_*o*_: the length of stretched myocardial fibers) refers to deformation of myocardial fibers, whereas LV EF refers to volumes and is therefore also dependent on ventriculo-arterial coupling [2]. Consequently, the fluid expansion induces changes in arterial elastance which may have more impact on EF than on GLS. Moreover, in the case of acute hypovolemia, as previously discussed, falsely elevated LV EF may be encountered due to a end-systolic collapse of the left ventricular. Therefore, after fluid loading and correction of left ventricular collapse the LV EF may not systematically increase even in the case of fluid responsiveness and increased inotropy. Finally, the LV EF was measured by Simpson’s biplane method, and even if this is the reference technique, it is associated with a non-negligible inter- and intraobserver variability which could have masked the LV EF modification.

Based on our study, the influence of preload on the strain values measured is critical to consider. Indeed, for preload-dependent patients, a low absolute value of GLS can lead to erroneous diagnoses of systolic dysfunction. As compared with previous studies evaluating strain in septic shock, our study sheds a new light on this technology [34–37]. Pathological strain values detect early myocardial dysfunction. However, it may not be relevant to assess systolic function in patients with unstable hemodynamic conditions. Therefore, in these patients, a low GLS value should push the clinician to question first a preload dependency and then a myocardial dysfunction. Before any strict evaluation of LV systolic function and before starting any inotropic treatment, hypovolemia should be ruled out.

In line with our previous study [13], the success rate of strain measurement was 82%, matching with cardiology studies [38]. However, in the critically ill patient, the success rate can drop to 50% [30]. In this regard, the use of a single apical 4-chamber view could facilitate the diffusion of speckle-tracking use in critically ill patients. Indeed, in our cohort, GLS (the average of the two, three and four apical views) and longitudinal strain of the apical four-chamber view only were strongly correlated.

Our study has several limitations of which we are aware. It is a single-center study. A single operator performed all of the measurements. Therefore, we could not assess the interoperator variability. Our resuscitation protocol left the choice of fluid solution to the prescribing physician. The albumin was administered over 30 min and the crystalloid over 60 min. These are indeed longer durations than recommended. Future studies should consider the association of this value and GLS.

## Conclusion

Our study showed that GLS was influenced by fluid resuscitation in preload-dependent patients. Interestingly, values shifted from pathological to normal after fluid resuscitation. In the critically ill patients, the assessment of systolic function by 2D-strain needs prior evaluation of preload dependency, in order to adequately interpret this variable. GLS variations after an increase in venous return could be a marker of the patient position in the Frank–Starling curve. Future studies evaluating the interest of GLS for guiding fluid resuscitation are required.

## Abbreviations

LV EF: left ventricular ejection fraction
LV: left ventricle
LVTDV: left ventricular tele-diastolic volume
LVSTV: left ventricular tele-systolic volume
LVOT VTI: left ventricular outflow track velocity–time integral
Δ LVOT VTI: variation of left ventricular outflow track velocity
PLR: passive leg raising
GLS: global longitudinal systolic strain
S4C: four-chamber systolic strain
S3C: three-chamber systolic strain
S2C: two-chamber systolic strain
